# Bioimpedance as an alternative tool for subjective, visual scoring of a prevalent ham quality defect

**DOI:** 10.2478/joeb-2024-0008

**Published:** 2024-06-28

**Authors:** Sisay Mebre Abie, Paweł Suliga, Bjørg Egelandsdal, Daniel Münch

**Affiliations:** 1 Faculty of Ecology and Natural Resource Management, Norwegian University of Life Sciences, 1432 Ås, Norway; 2 Faculty of Chemistry, Biotechnology and Food Science, Norwegian University of Life Sciences, 1432 Ås, Norway; 3 Animalia, Norwegian Meat and Poultry Research Center, 0513 Oslo, Norway

**Keywords:** Bioimpedance, subjective visual scoring, ham, meat defects

## Abstract

The detection of meat quality defects can involve both subjective and objective methods. PSE-like meat is linked to a common pork defect and can be caused by rapid post-mortem damage of muscle fibers. This damage can again be linked to various factors, such as a low ultimate pH or a higher slaughter weight. PSE-like defects are characterized by discoloration, structural damage, and excessive moisture loss. However, the lack of suitable instrument-based methods makes the detection of PSE-like defects difficult, and subjective methods typically suffer from poorer reproducibility. The objective of this study was to establish how subjective visual evaluation correlates with electrical impedance spectroscopy and with traditional quality parameters. To do so, visual scoring was performed together with measurements of bioimpedance, color, and pH in two ham muscles (Adductor, Semimembranosus) for 136 animals 24-hours post-mortem. When comparing with visual scoring, Pearson correlation analysis shows the strongest correlation for bioimpedance (*P_y_*, r = −0.46, R^2^ = 21%), followed by pH_u_ (r = 0.44, R^2^ = 19%). When using all five quality measures, i.e., *P_y_*, pH_u_, and CIELAB L^*^ a^*^ b^*^, the multivariate regression model had a prediction error of 0.76 for the visual scores. This was close to the error describing the subjective bias of visual scoring, more specifically the prediction error between the two observers (0.85). In all, *P_y_* showed the strongest correlation among instrument-based quality tests and alone may be used for predicting pork ham structural defects, i.e., as an instrument-based alternative for subjective, visual scoring. However, an instrument that combines *P_y_* with pH and/or L*a*b* would improve the prediction of PSE-like quality defects.

## Introduction

For the meat industry, reliable meat quality monitoring is of fundamental importance for profitability and consumer satisfaction [1]. Severe quality defects in meat are typically associated with unwanted attributes such as unacceptable appearance (pale color), reduced tenderness and juiciness or structural disintegration [2, 3]. The latter is especially relevant for pork, where “destructured” meat poses a challenge to high-quality ham production [4]. Destructured meat resembles typical features of PSE (Pale, Soft, Exudative) meat, which has been a global challenge for the pork sector in the last few decades [5]. The defect has been linked to various factors, such as rapid post-mortem pH decline, specific mutations, oxidative stress, and enhanced apoptotic processes [6–9]. While efforts have been made to eradicate mutations that can cause the defect [5], the prevalence of severe pork quality defects can be as high as 20 and even 50% in several European countries [3, 10, 11]. The terms PSE-like and destructured meat are often used interchangeably and describe a syndrome where several quality loss features coincide, i.e., color-, texture-, and pH anomalies with tissue disintegration and very low water holding capacity [12, 13]. Especially, the latter two features render PSE-like loin or ham unsuitable for costly curing processes. This is because the loss of these technological qualities often results in final products that fall apart when sliced and, hence, cannot be sold [3]. To at least reduce the financial burden of PSE-like pork, there is a need for objective test methods that can be used to detect and sort raw ham or loin with defects, before entering the costly processing streams for dry and wet curing.

Traditional methods for PSE-classification have been based on pH, color, and drip loss testing or on visual evaluation [14–18]. Yet, such PSE meat defect classification systems are not consistent among authors and are typically made for specific muscles, most often loin and less often ham muscles. Additionally, these methods have limitations in detecting specific quality features and may be less suited to predicting the prevalent structural defects that are typical for PSE-like ham [19]. Apart from PSE detection, other technologies like ultrasonic spectral analyses, X-ray measurements, and optical spectroscopy have been proposed as alternative quality assessment tools for pork meat quality [20–23]. In the absence of accessible instruments for objective measurements in meat-cutting plants, subjective, visual evaluation can offer a low-cost approach. Several subjective visual guidelines have been developed for pork defect evaluation [24–26]. As with other subjective tests, however, these methods have short-comings, e.g., a need for experienced users and poorer reproducibility compared to instrument-based testing. In addition, subjective color evaluation can also be biased by context, i.e., by different lighting, and also can be prone to day-to-day variations because of imperfect color memory [27].

Bioimpedance spectroscopy can offer a more reproducible, inexpensive, and fast method for testing diverse technological qualities of meat. Studies suggest the use of bioimpedance-based testing, particularly by using the *P_y_* parameter for several quality features, including tissue damage caused by freezing and thawing [28–30]. *P_y_* is an impedance meat quality parameter calculated from the extracted Cole parameters obtained by fitting measured impedance data to a Cole model [31, 32]. It has been previously shown that reduced bioimpedance response, and hence lower *P_y_* values, correlate with low pH and pale meat color. Low *Py* was also shown to be linked to microstructural damage in meat [21, 30, 32, 33]. In addition, bioimpedance spectroscopy was proposed as an indirect measurement of enhanced drip loss, where fluids leave the intracellular space and form larger drip channels [13, 34, 32]. We have previously shown how bioimpedance response is linked to other typical features of PSE-like ham, including low pH, pale color, and increased drip loss. However, studies are needed to directly benchmark bioimpedance spectroscopy against an established reference for PSE-like ham defects.

To this end, we studied the relationship between objective, instrument-based tests, including bioimpedance spectroscopy, and a subjective, visual quality test method. The visual test was based on an established reference for PSE-like ham defect evaluation. This allowed for scoring the severity of tissue and color defects [24], and in the following will be referred to as visual DES scoring. We asked how the different instrumental test methods predicted the visual DES score and how such prediction can be improved by establishing multivariate prediction models.

## Materials and methods

**Fig. 1.**
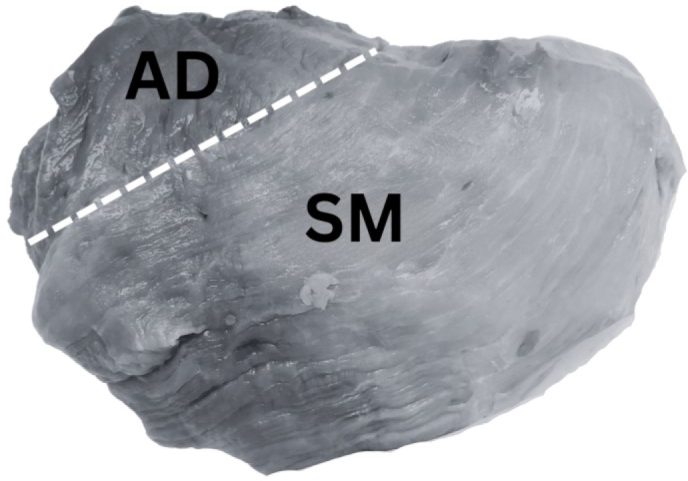
The two different muscles, for which bioimpedance, ultimate meat pH and CIELAB color values were measured. AD: adductor muscle, SM: semimembranosus muscle.

Pork (*Sus scrofa domesticus*) ham muscle samples from the semimembranosus muscle (SM), and the adductor muscle (AD, Fig. 1) were collected in October and November 2021 from a total of 136 animals. Pigs were slaughtered at one facility in Norway on five different days. Each day, we collected 25 to 36 samples at the cutting line, approximately 24 hours post-slaughter. To obtain samples that may represent a wide quality range, we opted for a heterogenous sample population, which included different producers and, hence, breeds as well as different farming types (free-range and conventional farming). Norwegian pigs typically have genetics that combine two or more of the following breeds: Landrace, Duroc, Z-line, or Hampshire.

The ham cuts were tested using subjective visual scoring and objective, instrumental methods. The latter included bioimpedance spectroscopy and the two most widely used meat quality tests, i.e., pH- and color-testing. Performing also drip-loss testing was not feasible, as sampling for drip loss would have largely destructed the valuable ham cuts, and transporting the cut up, smaller pieces to a laboratory would have caused drip to set in before controlled testing can commence. Measurements were made in the chilling room of the slaughterhouse. Common meat color coordinates (CIELAB/L*a*b* with L*: lightness, a*: redness, and b*: yellowness) were measured using a Konica Minolta Chroma Meter CR-400 (Konica Minolta Sensing INC, Japan; illuminant: D_65_, standard observer: 2°, diameter of the measurement area: 0.8 cm).

To allow for blooming, meat color was determined after the surface has been exposed to air for at least one hour. For calibration, a white ceramic calibration cap CR – A43 was used. The light source was a pulsed xenon lamp (Konica Minolta Sensing INC, Tokyo, Japan). The ultimate pH (pH_u_) was measured using a WTW pH 3110 (WTW, Germany), equipped with a BlueLine 21 pHT electrode. The pH-meter was calibrated before measurements using fresh buffer solutions with pH of 4.0 and 7.0. For each ham cut, pH and color values were recorded at two different anatomical locations, i.e., in the central region of each muscle (SM, AD; Fig. 1). The two muscles were included as there are known muscle-specific differences, e.g., in pH and color [3].

Next, bioimpedance was measured using a Zurich Instruments MFIA impedance analyzer (Zurich Instruments AG, Switzerland). Spectra were recorded for a frequency range from 10 Hz to 1 MHz, with 40 distinct frequency points and an applied voltage of 300 mV rms. A tetrapolar electrode setup with two signal-generating and two receiving electrodes was used. Electrode pins were made of stainless-steel with a diameter of 2 mm and a length of 12 mm. Pins were aligned in one row with 18 mm spacing between the middle, voltage pick-up electrodes, and 14 mm between the middle, voltage and the outer, signal-generating electrodes.

Shielded cables of 1 m length were used to connect the impedance analyzer and the electrode socket. Similar to pH and color testing, bioimpedance was measured at the two anatomical locations indicated in Figure 1. Two readings were recorded for each location of the electrode. The electrode was cleaned after every measurement.

Bioimpedance is a passive electrical property which is defined as the ability of the biological tissue to impede (oppose) the flow of electrical current. Bioimpedance can be measured by applying an electric excitation signal (either current or potential) and picking up the response of the tissue through electrodes, which convert the electronic charge to ionic charge and vice versa [33]. The complex electrical impedance produced by biological tissues is also called bioimpedance and can be expressed by the ratio of the voltage (V) and current (I).

$$Z=\frac{\text{V}}{\text{I}}$$

The complex electrical impedance of various organic tissues, including meat, results from contributions of both, tissue capacitance and conductance. This relation is expressed as,

Z=R+jX

where R is the resistance, or the real part of the impedance, and X is the reactance or the imaginary part of the impedance. As R and X are frequency dependent, impedance measurements provide a series of complex numbers which can be adjusted to a model that is described by the Cole mathematical expression (see [33, 36] for more details about these concepts).

$$Z={\text{R}}_{\infty }+\frac{{\text{R}}_{0}-{\text{R}}_{\infty }}{1+{\left(\text{jωτ}\right)}^{\propto }}$$

Here R_∞_ and R_0_ are the resistance or the electrical impedance modules at high and low frequency, respectively. τ is the characteristic time constant of the system corresponding to a specific angular frequency ω = 1/τ = 2πf_c_, where f_c_ is the characteristic frequency, which corresponds to the frequency at which the absolute value of the imaginary part of impedance is largest, and α is the distribution or shape adjustment and interaction parameter.

The specific relations of these parameters to structural changes in biological tissues are not completely clear. However, the normalized difference of *R*_0_ and R_∞_ is very sensitive to cellular integrity, and also to the ability of meat to bind water (“water holding capacity”). This normalized difference describes the response drop within a specific spectral band, the β-dispersion, and is termed the *P_y_* value [34]

$$P_{y}=\frac{{\text{R}}_{0}-{\text{R}}_{\infty }}{{\text{R}}_{0}}*100$$

The *P_y_* ranges for fresh, early post-mortem meat was previously described as ranging from 85 to 95, depending on the kind of meat [32, 34]. However, markedly lower values are also found for raw, unprocessed pork (> 24 h post-mortem [13, 28]. Due to freezing-related destruction of cellular structures, *P_y_* can further drop to very small values of *P_y_* < 10 [28, 37].

**Fig. 2.**
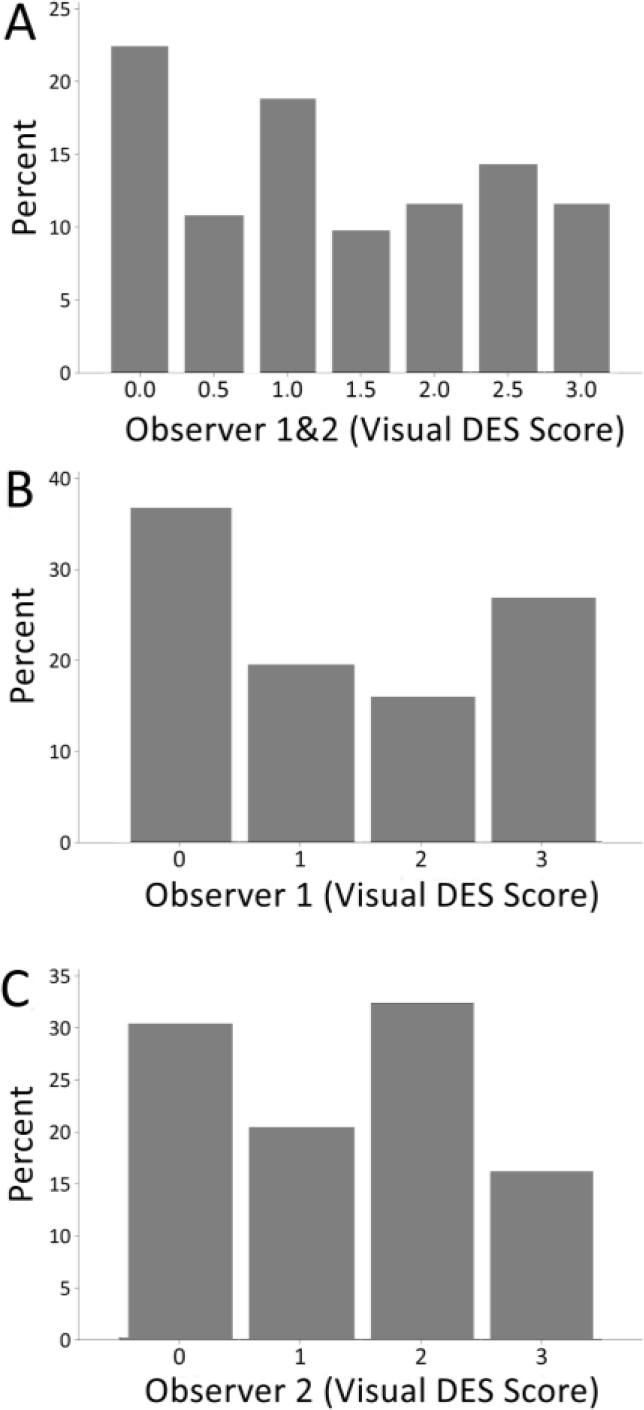
Visual DES defect scoring results of the two observers for N = 111 pork ham samples. A) the average visual scores for the two observers (note the 0.5 steps instead of integers). B, C) The distribution of the visual DES scores for the two observers separately. Both the average and individual score data sets show a sufficiently heterogeneous distribution, with all DES ranks being represented by a larger number of individuals and no heavily skewed distribution.

For visual evaluation, we adapted a ranking system for features related to tissue destruction (DES) and color established by IFIP [19, 24]. Briefly, the first step was to expose the inner part of the SM and AD muscles that were in close contact with the femur bone (*os femoris*), where most of the structural defects are typically spotted. Then a cut with a knife was done inside the muscle tissue for internal defect detection. Lastly, the degree of structural defect severity and Japanese color values were subjectively judged by two evaluators separately. During the study, the first 25 samples (day 1) were used for training and ‘normalizing’ the visual DES scoring among the two observers. In the training, both observers jointly evaluated the samples. For the remaining 111 samples, visual DES scores were given separately by the two observers, to also assess potential subjective, between-observer differences (see Fig. 2B, C for N = 111).

For testing relations between subjective DES scoring and instrument-based quality monitoring, average (N = 111 samples) and joint values (“training”, N = 25) of the two judges were used as a final DES score for each individual sample (N = 136). The observers gave DES scores according to the feature sets detailed in Table 1.

**Table 1.** The four-rank visual DES scoring scheme for destructured ham cuts, including the semimembranosus and adductor muscle. Scoring was based on evaluating structural disintegration and visual color (adapted from [19, 24]).

Score	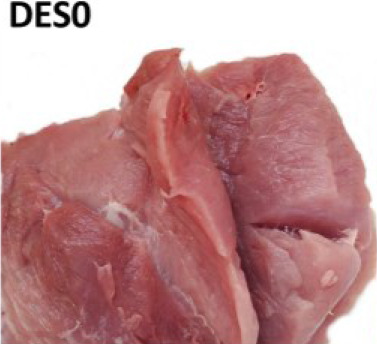	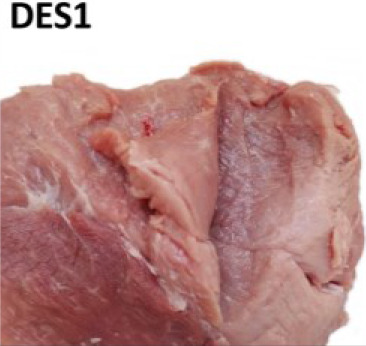	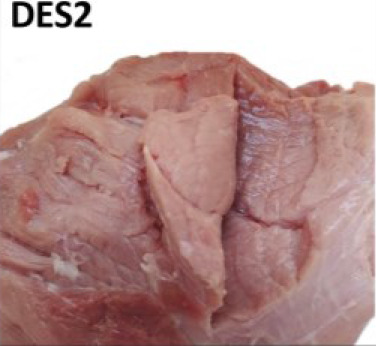	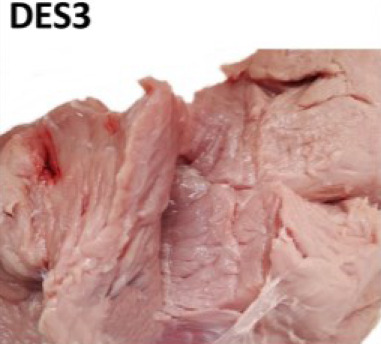
Visual colour	Reddish (> 3)	From pale to reddish (1 - 3)	Very pale (1 - 2)	
Muscle structure defect	Compact fibre structure	Absence of fibrillar structure in the affected area Destructured meat	Absence of fibrillar structure in the affected area Soft and doughy, destructured meat Fluid exudate	
Area affected	None	Small areas on the surface with single patches of destructured zones	More than 50% of both muscle areas Lesions beneath the surface	
Observations	No visible defects	Small, pale areas on the surface	Lesion less than approx. 2cm in depth	Lesion more than approx. 2cm in depth

Average scores were grouped into different meat defect ranks as follows:

- 0 to 0.5 = DES0: with no defects detected,
- 1 to 1.5 = DES1: mild,
- 2 to 2.5 = DES2: moderate,
- 3 = DES3: severe.

### Statistical analysis

The diverse sets of data were obtained from different analytical tools (electrical impedance analyzer, pH meter, colorimeter) and from visual scoring. Analysis of potential links among the DES score and quality parameters was conducted using the Pearson correlation coefficient (r). Calculations, correlation plots, and charts were generated with Minitab version 19 (LLC, Pennsylvania, USA). Significance levels were *P* < 0.05 for all tests. Stepwise regression analysis was performed with Minitab for generating multivariate models. MATLAB (version R2020a, The MathWorks Inc., Massachusetts, USA, 2020) was used to calculate prediction errors for the calculated models.

### Ethical approval

The research relates to the use of animal products and complies with all the relevant national regulations and institutional policies for the care and use of animals.

## Results

Subjective visual evaluation for PSE-like defects was performed by two independent evaluators following the visual scoring scheme (Table 1). Figure 2A shows the distribution of averaged visual scores for 111 ham samples. We found that all visual DES scores from 0 = no defect to 3 = severe defects were present in the sample population. This suggests that the sample population showed sufficient heterogeneity with samples ranking from normal to very poor quality, which is a prerequisite for exploring correlations with other test parameters.

There were detectable differences in subjective defect scoring among the two observers (compare Figs. 2B and C). Yet, r = 0.62 supported a strong correlation between the two observers’ DES scores. When calculating how good observer 1 DES scores can predict observer 2 data, we found a prediction error of 0.85 for a second-degree polynomial regression.

**Fig. 3.**
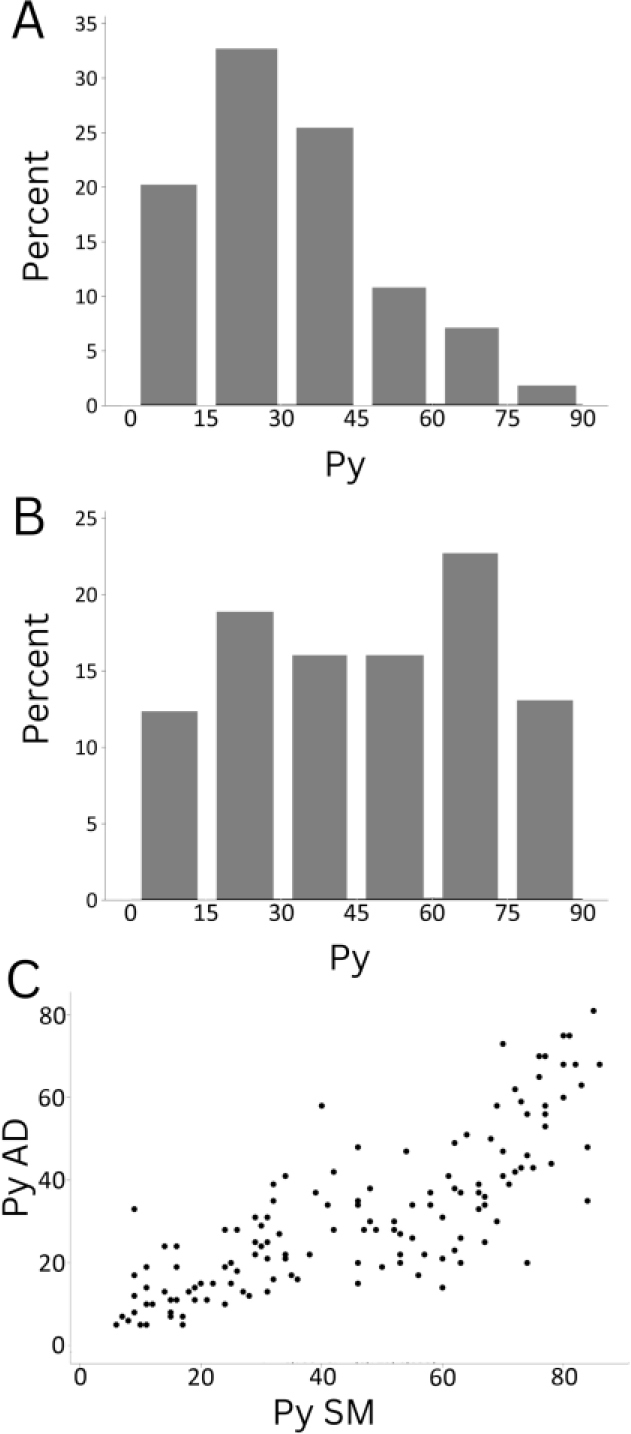
Bioimpedance response (*P_y_* parameter) distribution for two ham muscles, which were also included with the more global visual DES evaluation. A) *P_y_* parameter distribution for the AD muscle location. B) *P_y_* parameter distribution for the central SM location. C) Correlation plot of *P_y_* values scores for SM and AD testing indicating a strong correlation (r = 0.8).

We tested the bioimpedance response, based on the *P_y_* parameter, for the two muscles (Fig. 3) that were also assessed with visual scoring. We found that the distribution of *P_y_* values for the adductor muscle (AD, Fig. 3A) was shifted towards lower values, as compared to the semi-membranosus muscle (SM, Fig. 3B). However, *P_y_* data from the two muscles showed a strong correlation (Fig. 3C, r = 0.8, *P* < 0.05). This correlation and a shift towards lower *P_y_* in the AD – indicating a higher defect detection probability – prompted us to use the AD muscle for all subsequent analyses. This choice was also informed by a previous study, which found a strong gradient for PSE-like defects in the SM, where defects were limited to the ‘inner’ parts of the muscle, i.e., close to the femur [8, 23]. Therefore, intramuscular electrodes may sample a much more homogenous muscle volume, when testing the AD as compared to the SM.

**Fig. 4.**
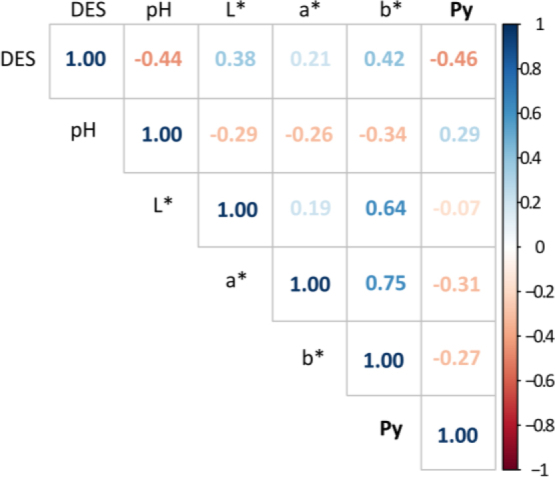
Correlation coefficients between visual destructured (DES) score and bioimpedance (BI) measurements, pH_u_, and CIELAB for the AD muscle. All shown correlations were found to be significant at (*P* < 0.05), except *P_y_* vs L* with *P* = 0.39.

We then tested how visual DES scores compare with *P_y_* and the traditional instrument-based measurements (pH_u_, CIE L*a*b* color values) in the AD muscle (Fig. 4). Correlation strength varied markedly among the parameters, with the strongest correlation found for *P_y_*. versus DES score (r = −0.461, *P* = 0.000), followed by pH_u_ (r = −0.44, *P* = 0.00), and moderate correlations, e.g., for color value a* (r = 0.21, *P* = 0.01). Correlations were typically only moderate for direct comparisons between the instrument-based tests ( *P_y_*, pH_u_, and CIE L*a*b*), which indicates that the different instrument-based variables cover different features of the quality defect.

This prompted us to ask, if the prediction of visual DES scoring can be improved by stepwise regression modelling and by including more parameters than just *P_y_* to predict visual DES scoring results.

We show that a linear multiple regression model (N = 136, see below) can provide a markedly higher correlation (r = 0.71) than we found for correlations of visual DES scores with only one instrument-based parameter only. The prediction error of the model was 0.76, i.e., the average deviation of the model prediction from the actual scoring data is less than 1 DES score point. Supplement 1 shows the model statistics, including the P values for each estimated coefficient.

$$\begin{array}{l} \text{Score}=-82.0+17.3\left({\text{pH}}_{\text{u}}\right)+2.33\left(L*\right)+0.854\left(a*\right)+0.211\\ \left(b*\right)+0.834\left(P_{y}\right)+0.0054\left(L{*}^{2}\right)-0.035\left(a{*}^{2}\right)-0.54({\text{pH}}_{\text{u}}\times \\ L*)+0.1165\left({\text{pH}}_{\text{u}}\times P_{y}\right)+0.0034\left(L*\times P_{y}\right). \end{array}$$

Lastly, we performed a stepwise multiple regression analysis of DES scores with *P_y_* and the traditional instrument-based tests also for the dataset that excludes training data, i.e., where the two observers gave a joint score (N = 111, see Materials and methods). We found that the model (not shown) gives a prediction error of 0.80, i.e., close to what we found for the model of the full N = 136 data set. Hence, prediction errors for both models were comparable, i.e., independent of using the full data set with joint scores and average DES scores or the subset with only average scores. Lastly, prediction errors for the multivariate models to predict visual DES scores were also comparable to the error calculated to assess how good subjective evaluation of one observer can predict the other observer’s scoring, i.e., a measure of subjective bias (see above and Fig. 2).

## Discussion

Using an established reference for subjective evaluation of pork quality defects (DES), we found the highest correlation for *P_y_*, followed by pH_u_, and then by CIELAB L*a*b* color variables. Our data therefore supports the use of bioimpedance testing for assessing PSE-like quality defects in pork. However, a model that combines *P_y_* with pH_u_ and L*a*b* markedly improved the prediction of subjective quality defect scoring (DES). Hence, our data suggest that combining bioimpedance and traditional PSE quality testing holds promise for developing instrument-based quality monitoring for a highly prevalent pork defect.

Correlations of subjective quality scoring with the different physicochemical quality variables (*P_y_*, pH_u_, and CIELAB L*a*b*) in our study ranged from moderate, with about r = 0.2, to almost r = −0.5. Other studies on pork quality defects show that also correlations among “objective”, physicochemical parameters can vary markedly [13, 32, 37–39]. Similarly, our previous study on PSE-like defects showed weak correlations, e.g., between *P_y_* and pH_u_ (r = 0.17), but higher correlations, e.g., for *P_y_* vs. drip loss (r = −0.31), *P_y_* vs. b* (r = −0.45), and for L* vs. drip loss (r = −0.24; [13]. In addition, comparing the previous results to the ones in the present study demonstrates that the correlations between specific quality parameters also can differ between studies. For example, in the present study, correlation coefficients for *P_y_* vs. pH_u_ and for *P_y_* vs. b* were r = 0.29 and r = −0.27, respectively, and hence, were higher for pH_u_ and lower for b* than in the previous study (compare Fig. 4 and [13]).

There are several likely explanations for different correlation strengths among different quality parameters and among different studies. For example, the well-known (“classic”) PSS or PSE defect is caused by a mutation that can generate a very distinct and easily detectable pH drop early post-mortem. In contrast, the “PSE-like defect” is a rather loosely defined term or syndrome, which is solely based on a set of detectable features, and is collectively applied to distinct, well-known defects (PSE/PSS, acid meat) as well as to defects with similar features (pale, soft, destructured), yet unknown causation. Depending on specific genetic or physiological predispositions, and also on causations linked to suboptimal pre- and post-slaughter practices, correlations between quality parameters may therefore vary within a test population or between studies. It is therefore conceivable that the relative expression of defect features and, hence, specific quality parameters, can be either dominated by pale discoloration (“color”) or destruction (“*P_y_*”) or by reduced water-holding capacity (“drip loss”) or can be highly severe for all common defect values. This would also support the use of multi-variable defect monitoring for the recently prevalent, likely multi-causal PSE-like defects, as opposed to previous defects that are routinely detected with, e.g., only early post-mortem pH testing.

Based on the above results and as before [13], we conclude that “*P_y_*” may carry some unique information, which is not expressed by standard quality parameters. In addition, each individual test method likely conveys some unique information related to ham defects. Consequently, this would limit the maximum possible correlation strength between *P_y_* and subjective quality defect scoring as a reference, as *P_y_* is associated with structural disintegration and drip loss [21, 32, 40], while subjective evaluation also covers color information (“paleness”). Further limiting expected correlation strength with subjective data is that the predictor variable (*P_y_*) is continuous, while subjective scoring generates ordinal data with four ranks only. However, the suitability of using *P_y_* to monitor structural disintegration more directly is supported by the highest correlation between *P_y_* and subjective quality assessment, with a focus on macroscopic structural defects (Fig. 4).

Our data corroborates a previous bioelectric study, which proposed the detection of PSE defects through combining the *P_y_* parameter with pH data [32]. While the former 2003 study may likely have tested a population, in which the “classic” PSE/PSS was prevalent, the study suggested that also the – then common – pH-based testing can be improved by including a more direct indicator of structural defects, i.e., a reduced *P_y_*. We here extend on this by including additional quality parameters (CIE L*a*b* values) in our prediction model for data on decidedly heterogenous pig population, for which PSE/PSS defects are unlikely as the respective mutation has been removed from Norwegian pig populations and was not found in a recent mutation mapping study (personal communication). In addition, we have previously demonstrated how multivariate (PCA-based) pork quality classification can help reveal protein abundance patterns that were specific for muscle tissue from meat classified as PSE-like. The multivariate detection strategies used by Pliquet et al. [32] and our studies differ therefore from studies using only one ‘defect feature’, e.g., BI-based parameters, such as impedance values for specific frequencies, impedance changes within a frequency band or phase angle [29] and references therein. Notably, therefore, our stepwise regression model can predict the average subjective data (Fig. 2A) to about the same prediction error we calculated to describe subjective bias, more specifically the differences between the two observers (Fig. 2B, C), respectively. This is despite inevitable bias or “noise”, inherent to subjective sensory scoring (see next section).

Follow-up studies are needed to test and improve the multivariate model by including larger data sets, also from other pig populations. In addition, applied instrument-based PSE-like detection classification strategies must be developed, in particular for segmenting continuous output data into binary (defect/no-defect) or ordinal ranking data (no/moderate/severe defect).

Subjective defect scoring can be a relatively cheap, low-barrier tool to classify raw pork meat and prevent cuts with severe quality defects from entering pricey and long-lasting processing streams, such as curing. However, subjective scoring schemes, including the one we used here, can suffer from insufficient normalization or reproducibility. Specifically, color perception can change in cutting plants with windows such as the one we performed our tests at in autumn, a period with rapidly changing daylight conditions. In addition, subjective bias inherently affects reproducibility, as we demonstrate with Figure 2. We cannot exclude that performing more tests over a longer period of time may have gradually reduced inter-individual observer differences. However, assessing pork defects involves haptic evaluation, visual color, and pattern of a cut with a relatively complex muscle anatomy [41]. Also, unlearning from visit to visit, and changed perception with context changes, i.e., with meat from different cutters, cannot be completely overcome in our or similar studies. However, for real-life applications, different evaluation (“perception”) by different cutting plants, cutters or sensors might contribute most to reduced reproducibility in industrial settings. Together, all this makes subjective scoring a typically less reliable reference standard for developing instrument-based detection tools. However, while it would be preferable to improve the detection capacity of novel tools by using instrument-based data as a standard, there is no unified physicochemical standard for PSE-like defects established yet [see [13] and references therein].

In addition to physiological and likely genetic variation among samples with PSE-like defects (see above), also instrument-specific challenges can reduce reproducibility and correlation-strength between different quality parameters. Specifically, meat pH is very location dependent and can vary markedly even in the same muscle and with measurements taken close to one another. This can add to the technical variation, that is known for measuring different muscles within the same individual (compare Fig. 3 for *P_y_*). By directly comparing the location dependency of pH and *P_y_* within individual meat cuts, and by measuring N = 48 pig loin samples from boars, we recently found a higher correlation for replicating *P_y_* measurements within meat cuts (r = 0.97) than for replicating pH data (r = 0.58, data not shown). Further, while pH and color measurements are sensitive to, e.g., temperature variation and oxidative state, respectively, bioimpedance measurements can be also confounded by temperature or sample dimension [28, 36]. While an effect of fiber orientation in relation to positioning of the tetrapolar electrode setup is conceivable, we detected such an effect only for chicken meat [42] but not for pork loin [28]. In conclusion, this suggests that approaches for bioimpedance-based PSE-like should include means to control for relevant confounding factors, e.g., by temperature compensation and by requiring a minimum sample size to avoid bias by *P_y_* variation in smaller samples that are close to the dimension of the electrode setup.

## Conclusion

Our study shows how subjective pork defect scores can be predicted by bioimpedance response (*P_y_*) and other standard quality variables. *P_y_* had the highest prediction capacity and alone may inform about the PSE-like quality state of ham samples. This supports bioimpedance to be a useful tool to assess and study pork defects, in particular if tissue disintegration is to be monitored. However, an instrument that combines *P_y_* with pH and/or L*a*b* may improve the prediction of PSE-like quality defects in pork.

## Figures and Tables

**Supplementary Table 1.** Estimated coefficients of the model statistics with P values.

	Estimate	SE	tSTAT	P - Value
**(Intercept)**	−82.022	74.573	−1.0999	0.274
**pH_u_**	17.343	13.121	1.3217	0.189
L*	2.3323	1.5671	1.4883	0.139
a*	0.85357	0.38534	2.2151	0.029
b*	0.21135	0.089393	2.3643	0.02
***P_y_***	−0.83391	0.27336	−3.0506	0.003
pH_u_ x L*	−0.54006	0.2669	−2.0235	0.045
**pH_u_ x *P_y_***	0.11651	0.045719	2.5484	0.012
L* x *P_y_*	0.0033977	0.0011399	2.9807	0.003
*L* ^*2^	0.0054231	0.0029521	1.837	0.069
*a* ^*2^	−0.035111	0.013828	−2.5392	0.012

## References

[1] Troy D.J., Kerry J. (2010). Consumer perception and the role of science in the meat industry. Meat science.

[2] Salas R.C.D., Mingala C.N. (2017). Genetic factors affecting pork quality: halothane and rendement napole genes. Animal biotechnology.

[3] Eliášová M., Kameník J., Saláková A., Pavlík Z., Pospiech M., Bohuslava T. (2017). The effect of PSE and non-PSE Adductor and Semimembranosus pig muscles on the occurrence of destructured zones in cooked hams. Journal of Food Quality.

[4] Theron L., Sayd T., Chambon C., Vautier A., Ferreira C., Aubry L., Ferraro V., Sante-Lhoutellier V. (2020). Toward the prediction of PSE-like muscle defect in hams: Using chemometrics for the spectral fingerprinting of plasma. Food Control.

[5] Barbut S., Sosnicki A., Lonergan SM., Knapp T, Ciobanu Daniel C, Gatcliffe L., Huff-Lonergan E., Wilson EW. (2008). Progress in reducing the pale, soft and exudative (PSE) problem in pork and poultry meat. Meat Science.

[6] Franck M., Bénard G., Fernandez X., Barbry S., Durand P., Lagant H., Monin G., Legault C. (1999). Observations préliminaires sur le jambon déstructuré. Description du phénomène et étude de quelques facteurs de variation. Journées de la recherche Porcine en France.

[7] Le Roy P., Elsen J.M., Caritez JC., Talmant A., Juin H., Sellier P., Monin G. (2000). Comparison between the three porcine RN genotypes for growth, carcass composition and meat quality traits. Genetics Selection Evolution.

[8] Théron L., Sayd T., Chambon C., Vénien A., Viala D., Astruc T., Vautier A., Santé-Lhoutellier V. (2019). Deciphering PSE-like muscle defect in cooked hams: A signature from the tissue to the molecular scale. Food chemistry.

[9] Franck M., Figwer P., Poirel M. (2000). CR des 8ème Journées des Sciences du Muscle et Technologies de la Viande.

[10] Balac D., Bazin C., Le Treut Y. (1998). Research of the factors able to influence the appearance of the syndrome of structureless hams. Polish Journal of Food and Nutrition Sciences.

[11] Minvielle B., Le Strat P, Lebret B, Houix Y, Boulard J, Clochefert N (2001). Viandes déstructurées, Situation dans cinq abattoirs de l’Ouest de la France: facteurs de risque et proposition d’un modèle. Caractérisation colorimétrique, biochimique et histologique. Journées de la Recherche Porcine.

[12] Hugenschmidt G., Hadorn R., Scheeder M. R., Silacci P., Scherrer D., Wenk C. (2010). The effects of early post-mortem pH and ultimate pH on level and amount of destructured zones in cooked cured hams. Meat Science.

[13] Suliga P., Abie S. M., Egelandsdal B., Alvseike O., Johny A., Kathiresan P., Münch D. (2022). Beyond standard PSE testing: An exploratory study of bioimpedance as a marker for ham defects. Meat Science.

[14] Garrido M., Pedauye J., Banon S., Laencina J. (1994). Objective assessment of pork quality. Meat Science.

[15] McDonagh C., Troy D., Kerry J., Mullen A. (2005). Relationship between the subjective and objective assessment of pork M. semimembranosus and classification of further processed pork quality. Food science and technology international.

[16] Brown S. (1992). A note on the use of subjective methods for assessing pig meat quality on the slaughterline. Meat Science.

[17] Forrest J.C. (1998). Reciprocal Meat Conference.

[18] Singham P., Birwal P., Yadav B. (2015). Importance of objective and subjective measurement of food quality and their inter-relationship. Journal of Food Processing & Technology.

[19] Vautier A. (2014). Les viandes déstructurées (Cahier du Mémento viandes et charcuteries). In IFIP-Institut du porc.

[20] Damez J.-L., Clerjon S. (2008). Meat quality assessment using biophysical methods related to meat structure. Meat science.

[21] Egelandsdal B., Abie S. M., Bjarnadottir S., Zhu H., Kolstad H., Bjerke F., Martinsen Ø. G., Mason A., Münch D. (2019). Detectability of the degree of freeze damage in meat depends on analytic-tool selection. Meat Science.

[22] ElMasry G., Sun D.-W. (2010). Hyperspectral imaging for food quality analysis and control.

[23] Theron L., Sayd T., Chambon C., Vautier A., Ferreira C., Aubry L., Venien A., Viala D., Astruc T., Ferraro V. (2020). Toward the Prediction of the PSE-Like Muscle Defect in Cooked Hams. Meat and Muscle Biology.

[24] Vautier A., Boulard J., Bouyssière M., Houix Y., Minvielle B. (2008). Prediction level of meat quality criteria on “PSE-like zones” defect of pork’s ham.

[25] Busboom J., Reeves J. (1997). Japanese meat grading. Washington State University Pullman, WA.

[26] Maignel L., Fortier M.-P., Lambert P., Riendeau L., Wyss S., Sullivan B. (2012). Defining carcass and meat quality standards for Canadian pork: Meat colour.

[27] Eagerman B., Clydesdale F.M., Francis F.J. (1977). Determination of fresh meat color by objective methods. Journal of Food Science.

[28] Abie S.M., Martinsen Ø.G., Egelandsdal B., Hou J., Bjerke F., Mason A., Münch D. (2021). Feasibility of using electrical impedance spectroscopy for assessing biological cell damage during freezing and thawing. Sensors.

[29] Zhao X., Zhuang H., Yoon S.C., Dong Y., Wang W., Zhao W. (2017). Electrical impedance spectroscopy for quality assessment of meat and fish: A review on basic principles, measurement methods, and recent advances. Journal of Food Quality.

[30] Pliquett U. (2010). Bioimpedance: a review for food processing. Food engineering reviews.

[31] Ayllon D., Seoane F., Gil-Pita R. (2009). 2009 Annual International Conference of the IEEE Engineering in Medicine and Biology Society.

[32] Pliquett U., Altmann M., Pliquett F., Schöberlein L. (2003). Py - a parameter for meat quality. Meat Science.

[33] Martinsen Ø.G., Grimnes S. (2014). Bioimpedance and bioelectricity basics.

[34] Pliquett F., Pliquett U. (1999). Stress action on biological tissue and tissue models detected by the Py value. Annals of the New York Academy of Sciences.

[35] Cole K.S. (1940). Cold Spring Harbor symposia on quantitative biology.

[36] Osen D.E., Abie S. M., Martinsen Ø. G., Egelandsdal B., Münch D. (2022). Bioimpedance-based authentication of defrosted versus fresh pork at the end of refrigerated shelf life. Journal of Electrical Bioimpedance.

[37] Byrne C., Troy DJ, Buckley DJ (2000). Postmortem changes in muscle electrical properties of bovine M. longissimus dorsi and their relationship to meat quality attributes and pH fall. Meat Science.

[38] Najar-Villarreal F., Boyle Elizabeth AE, Vahl Christopher I, Kang Qing, Houser Terry A, Gonzalez John M, Amamcharla Jayendra, Vega Daniel, Kastner Justin J, Cox M Keith (2021). Correlation of bioelectrical impedance with freshness quality attributes of beef longissimus lumborum steaks. Meat and Muscle Biology.

[39] Gjerlaug-Enger E., Aass L., Ødegård J., Vangen O. (2010). Genetic parameters of meat quality traits in two pig breeds measured by rapid methods. Animal.

[40] Kyle U., Bosaeus I., De Lorenzo AD., Deurenberg P., Elia M., Gomez JM. (2004). ESPEN guidelines for the use of BIA measurements----part I: review of principles and methods. Clin Nutr.

[41] Laville E., Franck M, Sidibé M, Sayd T, Bonny JM, Chazeix JF, Monin G (2003). Anatomical study of lesions in destructured ham. Sciences des aliments.

[42] Osen D. (2019). Differentiating between fresh-chilled and frozen-thawed chicken breasts and pork sirloins with bioimpedance, in Department of Physics.

